# Rapid Nanopore Sequencing to Identify Bacteria Causing Prosthetic Joint Infections

**DOI:** 10.3390/antibiotics14090879

**Published:** 2025-08-31

**Authors:** Hollie Wilkinson, Karina Wright, Helen S. McCarthy, Jade Perry, Charlotte Hulme, Niall Steele, Benjamin Burston, Rob Townsend, Paul Cool

**Affiliations:** 1 The Robert Jones and Agnes Hunt Orthopaedic Hospital NHS Foundation Trust, Oswestry SY10 7AG, UK; k.t.wright@keele.ac.uk (K.W.); h.s.mccarthy@keele.ac.uk (H.S.M.); j.k.l.perry1@keele.ac.uk (J.P.); c.hulme1@keele.ac.uk (C.H.); niall.steele@nhs.net (N.S.); paul.cool@nhs.net (P.C.); 2 School of Life Sciences, Keele University, Keele ST5 5BG, UK; 3 Sheffield Teaching Hospitals NHS Foundation Trust, Sheffield S5 7AT, UK; rob.townsend2@nhs.net; 4 School of Medicine, Keele University, Keele ST5 5BG, UK

**Keywords:** nanopore sequencing, prosthetic joint infection (PJI), bacterial identification

## Abstract

**Background/Objectives:** The diagnosis of prosthetic joint infection remains difficult. Microbiological cultures frequently have false-positive and false-negative results. This study investigates whether rapid nanopore sequencing can be used to aid the identification of bacteria causing prosthetic joint infection for more timely identification and treatment. **Methods**: Nineteen patients who had revision surgery following total joint arthroplasty were included in this study. Of these, 15 patients had an infected joint arthroplasty. All patients had joint fluid aspirated at the time of revision surgery. The DNA was extracted from these fluid aspirates, and rapid nanopore sequencing was performed using the MinION device from Oxford Nanopore Technologies. The sequencing data was trimmed to improve quality and filtered to remove human reads using bioinformatic tools. Genomic sequence classification was performed using the Basic Local Alignment Search Tool. The results were filtered by read length and sequence identity score. The European Bone and Joint Infection Society criteria were used as a standard to identify infected and not infected patients. Confusion tables were used to calculate accuracy and F1 score based on this criteria and the nanopore sequencing results. **Results**: Microbiological cultures and nanopore sequencing had an accuracy of 68% and 74%, respectively. However, combining both results predicted infection accurately in 94% of cases (F1 score 96%). **Conclusions**: Nanopore sequencing has the potential to aid identification of bacteria causing prosthetic joint infection and may be useful as a supplementary diagnostic tool.

## 1. Introduction

There are many established causes of PJI, with most cases occurring within 12 months of operation. These infections can be caused by contamination of the implant by direct contact or microorganisms in the air. PJI can also be caused by the spread of the infection to another site of the body, although this is less common [1]. Bacteria causing PJI often form biofilms, which are tight-knit communities of bacteria around the prosthesis surface. The bacteria in the middle of these biofilms can be difficult for the immune system and antibiotic treatment to reach, making effective eradication challenging.

The diagnosis of prosthetic joint infection (PJI) remains difficult, with no universally agreed standard. Several diagnostic criteria exist to diagnose PJI. These include the Musculoskeletal Infection Society (MSIS) and the European Bone and Joint Infection Society (EBJIS) criteria [1]. The BACH classification system is used to classify osteomyelitis and predict clinical outcomes. However, recently, the BACH classification has been extended to include PJI cases and was renamed JS-BACH [2].

The first indicators of PJI are usually clinical, aided by a blood test and radiological investigations [1]. Histology [3] and the alpha-defensin lateral flow test can aid in confirming infection. However, these investigations do not identify causative organisms, which are essential for guiding clinical management and antibiotic therapy [4].

Currently, the accepted method for identifying the causative bacterial species is microbiological cultures. This method has several limitations, including prolonged turnaround times and false-negative and false-positive results. False-negative results often occur due to insufficient sampling, previous antibiotic treatment, or microorganisms that are difficult to culture [1]. On the other hand, false-positive results are usually caused by contamination during sample taking or subsequent processing. The extended duration required for culture results delays the administration of targeted antibiotic therapy, thereby potentially jeopardising treatment. Delayed diagnosis can lead to prolonged patient suffering and increased treatment costs [5].

The treatment of PJI usually involves a combination of surgical intervention and antibiotic therapy. Surgical intervention is often the first step of treatment, and this is often when information is obtained regarding the causative organism and appropriate antibiotic therapy. This treatment combination aims to remove the infection and prevent its relapse. The success of treating PJI has varying success rates and can vary due to individual patient factors and the profile of the infection [1].

Nanopore sequencing is a third-generation genomic sequencing technology that is increasingly being utilised in infection diagnosis research. This technique facilitates a range of genomic sequencing methods, including whole-genome sequencing, 16S rDNA sequencing, and rapid sequencing [6,7].

This study aims to investigate whether rapid nanopore sequencing can be used to identify bacteria causing PJI from joint fluid aspirates for more timely identification and treatment.

## 2. Results

Samples from 19 patients were included for analysis, with 15 identified as being infected and 4 as not infected according to the EBJIS criteria and MDT decision. 

Using the JS-BACH criteria, two patients were classified as ‘J3’ and had limited therapeutic options. One of these patients had an amputation, and microbiological cultures yielded no growth. Nanopore sequencing identified *Pseudomonas aeruginosa*, which was later confirmed by further microbiological cultures (Table 1).

The results from nanopore sequencing are presented in a contingency table using the EBJIS criteria as the correct diagnosis (Table 2), enabling accuracy calculations.

Nanopore sequencing had an accuracy of 74%, precision (positive predictive value) of 92%, recall (sensitivity) of 73%, and F1 score (harmonic mean of precision and recall) of 81% (95% CI: 0.488–0.909).

The microbiological culture results from the same 19 patients are also presented in a contingency table using the EBJIS criteria as the correct diagnosis (Table 3).

Microbiological cultures had an accuracy of 68%, precision of 90%, recall of 67%, and F1 score of 77% (95% CI: 0.434–0.874).

The microbiological culture and nanopore sequencing results were combined; if one technique correctly diagnosed the infection, then that diagnosis was accepted. For example, if the bacterium in a patient is classified as infected according to the EBJIS criteria. If the bacterium was identified using nanopore sequencing but not microbiological cultures, then the sequencing diagnosis was adopted. The combined results from nanopore sequencing and microbiological cultures are shown in Table 4, and then the associated accuracy calculations were performed.

The results from combining nanopore sequencing and microbiological cultures had an accuracy of 94%, precision of 100%, recall of 93%, and F1 score of 96% (95% CI: 0.740–0.999).

### 2.1. False Positives

Rapid nanopore sequencing had one false-positive result, where *Escherichia coli* was identified in a patient without evidence of infection. This patient also had a negative alpha-defensin test and consequently was categorised as false-positive. One patient had a positive microbiological culture result but was identified as not infected during the MDT discussion, with *Staphylococcus epidermidis* deemed a contaminant.

### 2.2. False Negatives

Nanopore sequencing failed to identify the infective organism in four cases identified as infected by the EBJIS criteria, whilst microbiological cultures failed in five. By combining the results of the two methods, this was reduced to only one false-negative result. This patient had an ongoing PJI as per EBJIS criteria but was identified as not infected by rapid nanopore sequencing, and microbiological cultures were negative. The patient was treated with antibiotics leading up to surgery and had an ESR of 73 mm/hour and a CRP of 17 mg/L. The antibiotic treatment could explain the false-negative cultures and sequencing results for this patient.

### 2.3. Time to Obtain Results

**Figure 1:**
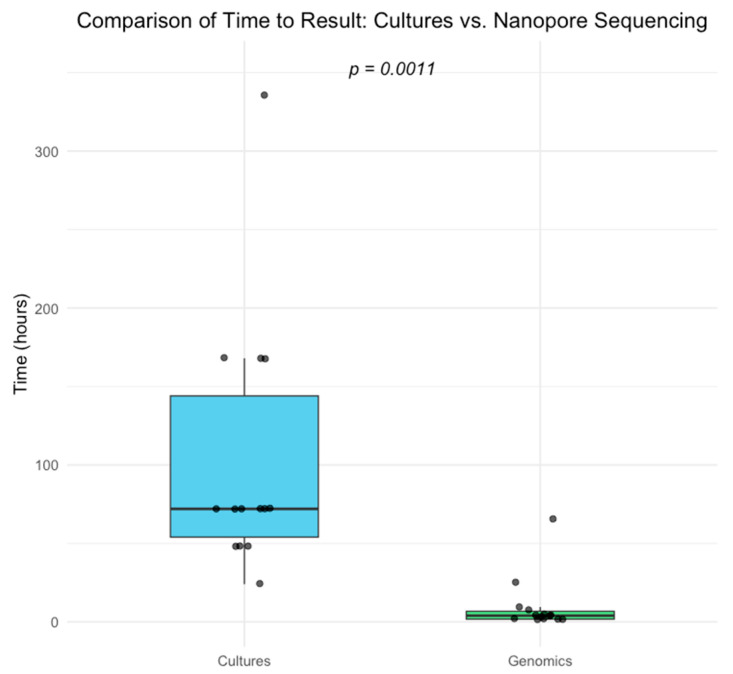
Boxplot comparing the time to result for samples classified as ‘infected’ according to the EBJIS criteria, using rapid nanopore sequencing (green) versus conventional microbiological culture (blue).

The time to obtain results for nanopore sequencing and microbiological cultures was compared for samples identified as ‘infected’ by the EBJIS criteria. For these 15 samples, the average time in hours to obtain results was calculated for both methods. The mean time for nanopore sequencing to obtain results was 9.8 h (IQR = 5), whilst the mean time for microbiological cultures was almost ten times that, at 97.8 h (IQR = 90, *p* < 0.001, Figure 1).

## 3. Discussion

The main advantage for the use of nanopore sequencing for the diagnosis of PJI is the reduced turnaround time and increased sensitivity of the results compared to microbiological cultures. In addition, tests such as an alpha-defensin lateral flow test have high costs and limited diagnostic information. Microbiological cultures generally have a turnaround time of 7–14 days, but nanopore sequencing has the potential to produce results within 24 h. This time saving could have implications with regard to reducing the length of hospital stays, treatment periods, and even the time frame between the first and second stages of a two-stage prosthetic exchange. This could lead to improved patient outcomes and reduced treatment costs, as the current costs associated with treating PJI are estimated at around EUR 100,000 in the NHS [8].

Nanopore sequencing has previously been used to diagnose infection successfully in hospital patients with lung infections [9]. In this study, the authors also achieved high sensitivity (96.6%) but had a reduced specificity of 42.7%. They did not analyse the results by combining nanopore sequencing and microbiological cultures, but using confirmatory PCR after results were obtained improved the specificity and sensitivity to 100%. Similar to this study, they were limited by the number of samples used for analysis (41 samples) [9].

The results of nanopore sequencing are comparable to microbiological cultures, with similar results and accuracy. However, combining both investigations improves the accuracy of the diagnosis of infection to 94%. Hopefully, with further development, this can be further improved.

### 3.1. Challenges

A major limitation of this study was the small sample size (*n* = 19), with only four patients identified as ‘not infected’ included in the analysis; these patients were also all from the same establishment. These factors may represent bias in the sample. In addition, the diagnosis of these patients was based only on the EBJIS criteria, which may not be the adopted diagnostic criteria at other sites.

The results obtained from nanopore sequencing in this study required careful interpretation, including comprehensive data processing and the removal of bacterial species detected but not associated with PJI. Some sequencing outcomes suggested the presence of potential ‘false positives’, complicating the accurate identification of pathogenic species and the question of whether these are polymicrobial infections or part of the normal flora. Given the high sensitivity of nanopore sequencing, which can sequence a single DNA strand, there is a risk of identifying bacterial species that are not clinically relevant to the infection. Additionally, contamination with a small number of bacteria may contribute to false identification. There is uncertainty around the ‘ground truth’ of the bacterial species causing PJI, as current methods of microbiological cultures are known to frequently produce both false-positive and false-negative results. This makes developing a single ‘gold standard’ diagnostic test difficult, as the ‘true result’ is never certain [10]. This may mean a combination of diagnostic tools should be used for diagnosing PJI.

Nanopore sequencing exhibits a higher error rate compared to other genomic sequencing methodologies [6]. Consequently, careful consideration was given to optimising data filtering strategies based on the identity score, E-value, and sequence length. These parameters must be set to account for errors inherent to nanopore sequencing and natural mutations occurring within a species over time. However, they must also be stringent enough to maintain analytical accuracy and prevent incorrect identifications. Preliminary investigations were conducted to determine threshold values. The classification results with a longer length had lower associated E-values, so only the length and identity score were used for filtering. No universally accepted standards exist for filtering nanopore sequencing data; as a result, data analysis remains a challenge in the application of nanopore sequencing.

The process of DNA extraction often requires a specific volume of sample input. The volume of joint fluid aspirate can vary depending on the patient. In cases of mild or low-grade infections, obtaining an adequate aspirate may be challenging, particularly before performing a revision procedure. This may necessitate performing a two-stage procedure so samples can be taken from the first stage for microbial identification. In such cases, a limited volume of fluid may be insufficient for DNA extraction, and tissue-based extraction may present additional challenges due to the high proportion of host cells. Consequently, the application of nanopore sequencing may be most useful in cases where a sufficient volume of aspirate can be obtained. In addition, to perform nanopore sequencing, a minimum DNA input quantity is required.

The confidence intervals presented above suggest that the precision of the accuracy values may be improved by increasing the sample size. The CI for the combined approach (0.740–0.999) is narrower and skewed toward higher values, suggesting better precision here, which could reflect either higher sensitivity/specificity or more robust results when combining methods.

### 3.2. Future Investigations

Further investigations will be useful to optimise the methodology. This may include investigations in the sample preparation technique, such as the biological depletion of host DNA or using alternative library preparation kits [11], as only the Rapid Sequencing Kit (SQK-RAD004, Oxford Nanopore Technologies, Oxford, UK) and 16S Barcoding Kit (SQK-16S114.24, Oxford Nanopore Technologies) have been used to date. In addition, the use of an alternative nanopore sequencing platform, such as the PromethION, may increase the speed and quality of data generation [11]. No host depletion was performed, and although this may be suboptimal for producing results, we focused on the speed of results, as host depletion techniques can be time-consuming and therefore were not used in this investigation. Furthermore, handling the sample more than necessary may increase the chance of sample contamination, leading to false identification.

Future work should include validation of these results for clinical practice. This may be performed by replicating the protocol at another site and comparing the results to see if similar accuracy can be achieved with similar patient cohorts. These results would need to be validated on a larger cohort of patients at multiple sites to consider clinical implementation of nanopore sequencing to identify bacteria associated with PJI. This may first require further work at this site to increase the sample size and still show similar results.

## 4. Materials and Methods

All patients included in this study had revision surgery following a joint replacement, where a sample of joint fluid aspirate was obtained. The study was approved by the Health Research Authority (reference 20/HRA/4857) and all patients gave informed consent.

A diagnosis of ‘infected’ or ‘not infected’ was determined following discussion of clinical assessment, blood tests, imaging, microbiology, and histology results at the infection multidisciplinary team meeting and according to the EBJIS criteria. Table 5 shows the clinical diagnoses using these criteria, considering the clinical presentation, imaging, biochemical test results, histological imaging and any microbiological culture results from previous samples.

### 4.1. Microbiological Culturing

Microbiological samples were processed in a category two cabinet to protect the sample from contamination and cultured in four different ways: anaerobic broth, aerobic broth, a standard agar plate and an agar plate enriched with horse blood. The standard incubation time was seven days, but incubation was extended to 14 days for aspirates from a suspected PJI. If there was insufficient sample to carry out all four cultures, only the aerobic and anaerobic broths were used (abolishing the agar plate cultures). On indication (i.e., slow-growing organism suspected), the culture time was extended to 21 days.

### 4.2. Nanopore Sequencing

All revision surgery was performed in an ultra-clean air theatre, where the fluid aspirates were collected in sterile sample pots. DNA was extracted using the MagAttract HMW Kit (Qiagen, Manchester, UK). The DNA was then quantified using a microplate reader (FLUOstar Omega, BMG Labtech, Ortenberg, Germany), and the purity was checked by calculating the A260/280 ratio. If the DNA library has an A260/280 of ~1.80, it was deemed suitable for nanopore sequencing [12].

Subsequently, the DNA was prepared with the rapid sequencing kit (SQK-RAD114, Oxford Nanopore Technologies) according to the manufacturer’s instructions and loaded onto the flow cell (version R9.4.1 or R10.4.1) to be sequenced with the MinION device (Oxford Nanopore Technologies). Sequencing time ranged from 15 min to 24 h, with a minimum accepted Q score of 8. Base calling was performed with Guppy [13]. Samples were excluded if there were fewer than 500 reads.

Sequencing of nuclease-free water was performed in the early stages of this research and identified marine organisms that are associated with microbial species previously associated with biological reagents, termed the ‘kitome’. These bacteria are not thought of as infection-causing or pathogenic, so they were easily filtered out. We cannot control contamination during sample collection, which is a limitation of the current use of microbiological cultures.

### 4.3. Bioinformatic Analysis

Adapters applied to the reads in the library preparation process were removed with Porechop (v2.0.4) and low-quality ends (10 nucleotides) were trimmed with Nanofilt (v2.8.0) [14,15]. Read quality was checked with FASTQC (v0.12.1) and mapped against the human reference genome (GRCh38) using Minimap2 (version 2.17) [16,17]. The unmapped reads were retained in fastq format with Samtools (version 1.21) [18].

Bacterial identification was performed with Basic Local Alignment Search Tool (BLAST) against the National Center for Biotechnology Information (NCBI) bacterial database [19]. Using R (version 4.4.2), the results were filtered to retain identifications with a minimum sequence length of 500 base pairs, a minimum identity score of 80, and an E-value of 0.01 [12]. The E-value represents the statistical significance of the alignment between the query and reference sequence. In R, only species identified as being associated with causing infections in humans were retained in the results using a predetermined list [12]. Following nanopore sequencing, a report was created containing the results and other clinical indicators of infection.

Continuous variables could not be modelled with a normal distribution and summarised with the median and interquartile range. Categorical variables are presented as proportions. Contingency tables were created to assess the agreement between clinical decisions and the results of nanopore sequencing/microbiological cultures. The accuracy, precision, recall, and F1 score were calculated for each table. The F1 score is the harmonic mean of the precision and recall.

Classifications were compared with the McNemar test, and a *p* value of less than 5% was deemed significant.

### 4.4. Data Analysis

The original BACH is a criterion designed to classify different aspects of bone osteomyelitis: bone involvement (B), antimicrobial options (A), coverage of the soft tissue (C), and host status (H). Each of these aspects is given a ranking of 1, 2, or 3, with 1 being ‘uncomplicated’, 2 being ‘complex’, and 3 being ‘limited options’ for that aspect of osteomyelitis [2]. This criterion was recently adapted for the classification of PJI by adding the criterion ‘J’ for PJI. Classification of J1 was made if there was no evidence of bone loss, loosening, or periprosthetic fracture; J2 if there was moderate bone loss, associated periprosthetic fracture or a non-primary type implant; and J3 if there was major bone loss, a custom implant, or custom/total bone replacement required for reconstruction.

The final results from nanopore sequencing were compiled in a clinical report (Supplementary Materials). The time to obtain results for nanopore sequencing and microbiological cultures was compared for samples identified as ‘infected’ by the EBJIS criteria, and a Wilcoxon signed-rank test was performed.

## 5. Conclusions

Overall, the results of this study are encouraging, and with further development, nanopore sequencing is likely to aid the identification of bacteria causing PJI as a supplementary tool and improve the speed of bacterial identification.

## Figures and Tables

**Table 1:** The JS-BACH criteria followed for classifying patients in this study (number one or ‘X’ represents ‘uncomplicated’, number two ‘complex’, and number 3 ‘limited options’ for the aspect of the identified PJI case) [2].

For PJI (JS)	Antimicrobial (BA)	Coverage (C)	Host Status (H)	Number Patients
J1	A1	C1	H1	3
J1	A1	C1	H2	3
J2	A1	C1	H1	2
J2	A1	C1	H2	3
J2	Ax	C1	H1	1
J2	Ax	C1	H2	1
J3	A2	C1	H2	1
J3	A3	C1	H2	1
			Total	15

**Table 2:** Contingency table comparing the result of nanopore sequencing with the EBJIS criteria classification (McNemar test, *p* = 0.371).

EBJIS	Nanopore Sequencing	
	Infected	Not Infected
Infected	11	4
Not Infected	1	3

**Table 3:** Contingency table comparing the result of microbiological cultures with the EBJIS criteria classification (McNemar test, *p* = 0.221).

EBJIS	Microbiological Cultures	
	Infected	Not Infected
Infected	10	5
Not Infected	1	3

**Table 4:** Contingency table comparing the result of combining nanopore sequencing with microbiological cultures with the EBJIS criteria classification (McNemar test, *p* = 1).

EBJIS	Sequencing + Cultures	
	Infected	Not Infected
Infected	14	1
Not Infected	0	4

**Table 5:** The clinical findings and analysis of the patients enrolled in this study.

Sample ID	CRP (mg/L)	ESR (mm/hr)	PMN (10 µg/L)	Culture	Bacterial Species	Clinical Notes
004	23	65	9.5	Positive	*S. aureus*, *Strepptococ cus*	Above-knee amputation
017	39	19	6.6	Negative	n/a	Clinically not infected, asthmatic
018	150	104	5.2	Negative	*Escherichia coli*	Persisting infection following TKR
020	42	77	6.2	Negative	*Escherichia coli*	Persisting infection following TKR
022	5	8	n/a	Negative	n/a	Clinically not infected
023	1	5	3.0	Negative	n/a	Clinically not infected
024	2	20	10	Negative	n/a	Clinically not infected
026	47	101	6.5	Positive	*E. faecalis*	Synovasure positive
028	68	47	9.2	Positive	*S. aureus*	Sepsis episodes
029	68	47	9.2	Positive	*S. aureus*	Sepsis episodes
031	36	49	3.8	Positive	*S. epidermidis*, *S. capitis*	Persisting infection following TKR
036	2	27	6	Positive	*S. anginosus, Anaerobes*	Poor host with many comorbidities
038	7	5	3.3	Negative	n/a	Second stage of revision
046	177	83	n/a	Positive	*S. aureus*	Persistent delayed-onset PJI
063	25	52	n/a	Positive	*S. aureus*	Persistent delayed-onset PJI
065	14	29	7.4	Negative	n/a	No clear clinical indication of PJI
074	17	73	5.5	Positive	*Staphylococcus*	First stage of two-stage revision
096	26	35	4.3	Positive	*S. capitis*	No comorbidities
098	27	34	5.5	Positive	*Pseudomonas*, *E. faecalis*	Amputation for infection, previous cultures all negative

CRP, C-Reactive Protein; ESR, Erythrocyte Sedimentation Rate; PMN, Polymorphonuclear Neutrophils.

## Data Availability

The datasets presented in this article are not readily available because the data are part of an ongoing study.

## Supplementary Materials

The following supporting information can be downloaded at: https://www.mdpi.com/article/10.3390/antibiotics14090879/s1, Figure S1: Nanopore Sequencing Report.

## Abbreviations

The following abbreviations are used in this manuscript:

MSIS	Musculoskeletal Infection Society
EBJIS	European Bone and Joint Infection Society
MDT	Multi-Disciplinary Team
ESR	Erythrocyte Sedimentation Rate
CRP	C-Reactive Protein
PCR	Polymerase Chain Reaction
NHS	National Health Service
ONT	Oxford Nanopore Technologies
BLAST	Basic Local Alignment Search Tool
PJI	Prosthetic Joint Infections
