# Supplementary figures and images for: Rapid Nanopore Sequencing to Identify Bacteria Causing Prosthetic Joint Infections
Source: Antibiotics (Basel). 2025 Aug 31;14(9):879. doi: 10.3390/antibiotics14090879 (PMC12466696; doi:10.3390/antibiotics14090879)

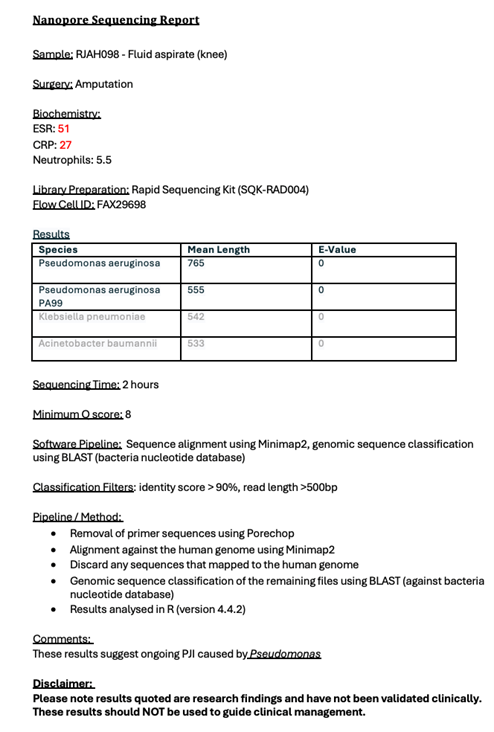

Supplement: Supplementary file 1 [file antibiotics-14-00879-s001.zip › antibiotics-3768573-supplementary.png]
